# Fluvoxamine stimulates oligodendrogenesis of cultured neural stem cells and attenuates inflammation and demyelination in an animal model of multiple sclerosis

**DOI:** 10.1038/s41598-017-04968-z

**Published:** 2017-07-07

**Authors:** Majid Ghareghani, Kazem Zibara, Heibatollah Sadeghi, Shima Dokoohaki, Hossein Sadeghi, Roya Aryanpour, Amir Ghanbari

**Affiliations:** 1 Cellular and Molecular Research Center, Faculty of Medicine, Yasuj University of Medical Sciences, Yasuj, Iran; 2 ER045, Laboratory of stem cells, PRASE, Biology department, Faculty of Sciences-I, Lebanese University, Beirut, Lebanon; 3 Medicinal Plants Research Center, Faculty of Medicine, Yasuj University of Medical Sciences, Yasuj, Iran

## Abstract

Multiple Sclerosis (MS) require medications controlling severity of the pathology and depression, affecting more than half of the patients. In this study, the effect of antidepressant drug fluvoxamine, a selective serotonin reuptake inhibitor, was investigated *in vitro* and *in vivo*. Nanomolar concentrations of fluvoxamine significantly increased cell viability and proliferation of neural stem cells (NSCs) through increasing mRNA expression of Notch1, Hes1 and Ki-67, and protein levels of NICD. Also, physiological concentrations of fluvoxamine were optimal for NSC differentiation toward oligodendrocytes, astrocytes and neurons. In addition, fluvoxamine attenuated experimental autoimmune encephalomyelitis (EAE) severity, a rat MS model, by significantly decreasing its clinical scores. Moreover, fluvoxamine treated EAE rats showed a decrease in IFN-γ serum levels and an increase in IL-4, pro- and anti-inflammatory cytokines respectively, compared to untreated EAE rats. Furthermore, immune cell infiltration and demyelination plaque significantly decreased in spinal cords of fluvoxamine-treated rats, which was accompanied by an increase in protein expression of MBP and GFAP positive cells and a decrease in lactate serum levels, a new biomarker of MS progression. In summary, besides its antidepressant activity, fluvoxamine stimulates proliferation and differentiation of NSCs particularly toward oligodendrocytes, a producer of CNS myelin.

## Introduction

Multiple sclerosis (MS) is an inflammatory disorder of the central nervous system (CNS), characterized by demyelination and axonal damage^1^. MS is also associated with considerable psychiatric problems, including depression and anxiety. Indeed, some studies reported more than three times higher prevalence of depression in MS patients^2–4^. Along with the common treatments, which act by modifying the inflammatory and demyelination situations, antidepressant drugs are also prescribed for management of depressive disorders, among which selective serotonin reuptake inhibitors (SSRIs) are most commonly used including *sertraline*, *citalopram*, *paroxetine*, *fluoxetine* and *fluvoxamine* ^5^.

In fact, SSRIs are considered as first line pharmacological treatment of moderate to severe depressive disorders in MS whereas the monoamine oxidase inhibitors (MAOIs) and tricyclic antidepressants (TCAs) act as second line treatments^6^. Some reports studied the use of antidepressant drugs in MS^7–10^, all of which indicated their beneficial effects regarding depression related symptoms. Indeed, very few studies investigated the potential immunosuppressive effect of *fluoxetine* ^11^ and *sertraline* ^12^ in animal models of MS. On the other hand, only one study in MS patients with major depression showed the efficacy of *fluvoxamine* ^8^. Recently, in relapsing-remitting MS with depression, Bayas and colleagues used, for the first time, the combination treatment of SSRI and *Fingolimod*, a confirmed and common therapy for relapsing MS, which showed improvement in depression condition of MS patients while disability remained unchanged^13^. Moreover, *fluoxetine* was shown to act as a differentiation factor for mouse embryonic stem cells (mESCs) into glial cell lineage^14^. It is worth noting that embryonic neural stem cells (eNSCs) are the more appropriate cells for studying therapeutic and neurotoxic effects in the CNS since they are self-renewing cells which can generate mature cells of all neural lineages, including oligodendrocytes, neurons and astrocytes^15, 16^.

In this study, effects of the antidepressant drug *fluvoxamine* (PubChem CID: 5324346) was investigated on rat embryonic NSC proliferation and differentiation *in vitro* as well as on pathological outcomes of inflammation and oligodendrogenesis in an animal model of acute MS *in vivo*.

## Results

### In vitro

#### The effect of fluvoxamine on NSCs viability and neurosphere frequency

**Figure 1:**
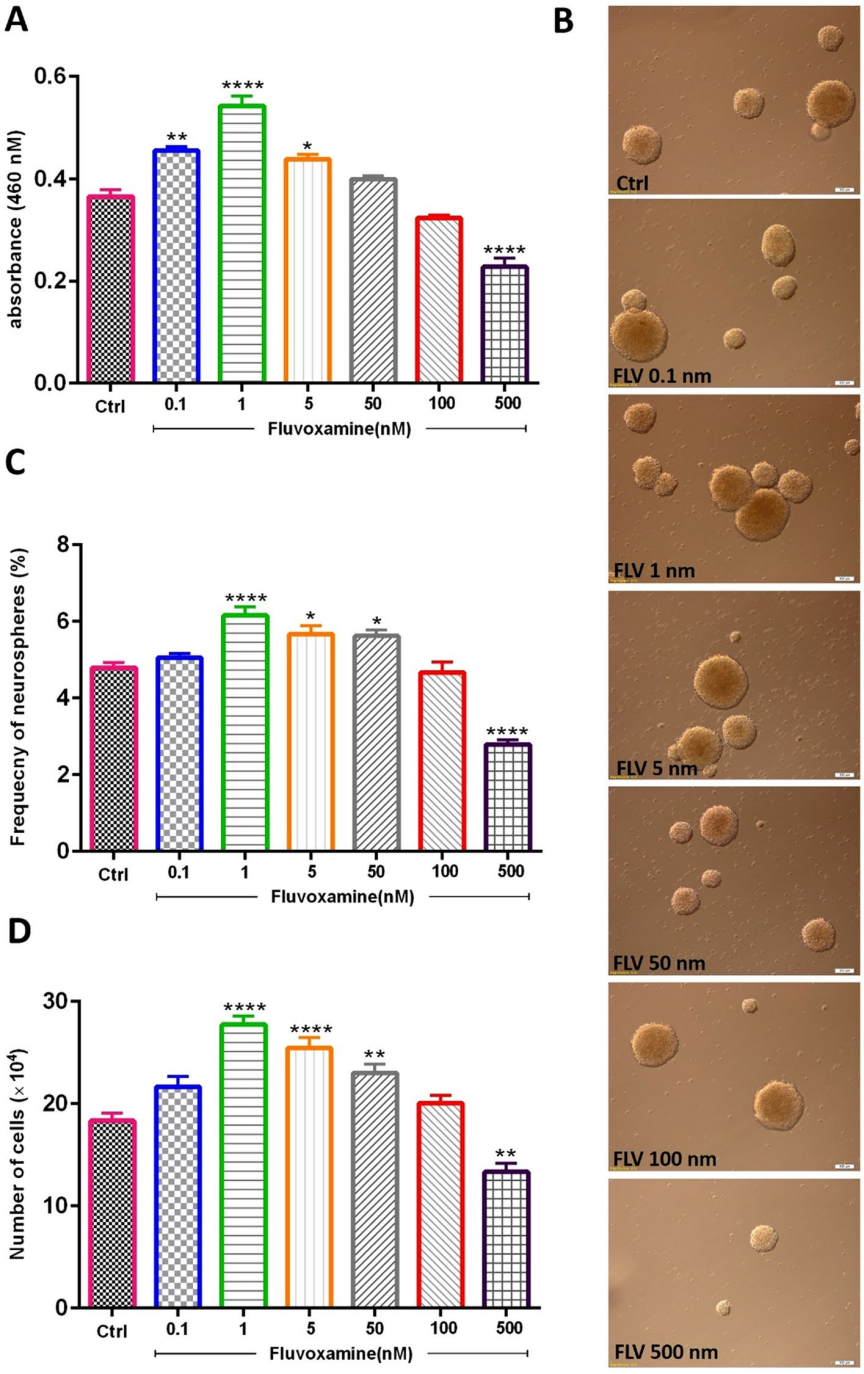
Effect of fluvoxamine on NSC viability and neurosphere formation *in vitro*. (**A**) The number of viable cells was evaluated using MTT assay. *Fluvoxamine* increased the number of viable cells as compared to the control group. Each bar represents the mean value of absorbance at 460 nm. (**B**) Representative images of neurospheres in the different groups. Scale bar = 100 μm (**C**) *Fluvoxamine* significantly increased neurosphere formation at 1, 5, and 50 nM, while it was toxic at 500 nM. (**D**) Cell counts obtained from neurospheres showed an increase of the mean cell number at 1, 5 and 50 nM. Data were expressed as mean ± SEM and each experiment included 15 replicates per condition (n = 15).

The effect of various concentrations of *fluvoxamine* (0.1, 1, 5, 50, 100 and 500 nM) on NSC viability was first evaluated using MTT assay. Results showed that low concentrations of *fluvoxamine* (0.1, 1 and 5 nM) significantly increased NSC viability (Fig. 1A). For instance, the optimal concentration of 1 nM *fluvoxamine* caused ~1.5-fold increase in cell viability, in comparison to controls (****p < 0.0001). In addition, NSCs formed neurospheres of various sizes with diameters ranging between 50 µm to >100 µm (Fig. 1B). Similarly, neurosphere formation frequency, which reflects the self-renewal capacity of NSCs, increased significantly at 1, 5 and 50 nM *fluvoxamine* concentrations with a peak at 1 nM (6.15 ± 0.23), in comparison to controls (4.78 ± 0.14) (****p < 0.0001; Fig. 1C). Finally, counting single cells obtained from the neurospheres also demonstrated a significant increase, similar to the neurosphere frequency, with a peak cell number at 1 nm of *fluvoxamine*, in comparison to controls (****p < 0.0001; Fig. 1D). These data suggested that while *fluvoxamine* might be toxic at 500 nM concentrations, it caused an increase in NSC proliferation at lower concentrations.

#### The effect of fluvoxamine on notch signaling

**Figure 2:**
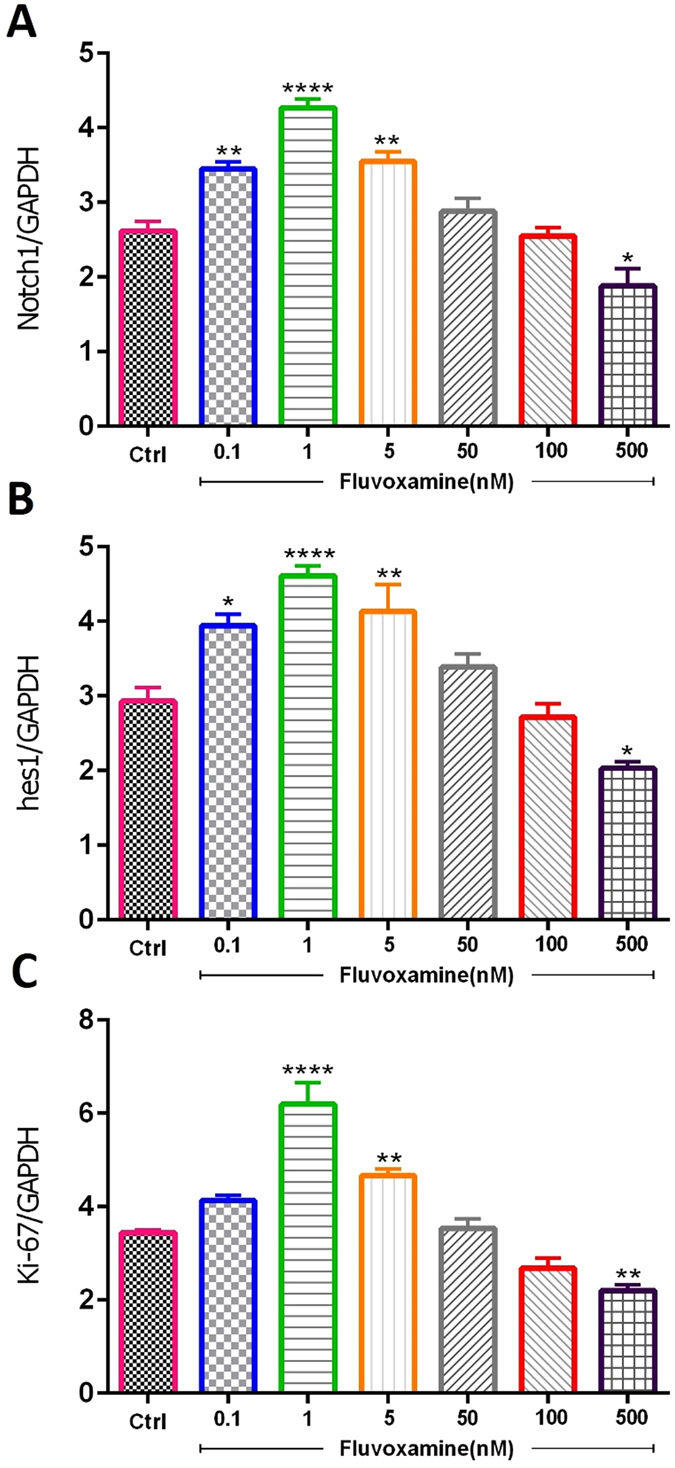
Effect of fluvoxamine on mRNA expression levels of Notch1, Hes1 and Ki-67 transcription factors. ENSCs were cultured with 0.1, 1, 5, 50, 100 or 500 nM concentrations of *fluvoxamine* for 5 days. Total RNA was prepared from each culture, cDNA synthesized and subjected to real-time PCR, using specific primers for Hes1, Notch1 or ki-67. GAPDH was used as an internal control. Each experiment included 5 replicates per condition (n = 5). The values are expressed as the mean ± SEM.

The effect of *fluvoxamine* on certain basic helix-loop-helix (bHLH) transcription factors, which play important roles in the proliferation and differentiation of NSCs, was then determined (Fig. 2). Indeed, some bHLH factors, such as Notch1 and Hes1, promote stemness and proliferation, while others, such as Mash1 and NeuroD, promote neuronal differentiation^17–19^. Treatment of NSCs with 0.1, 1 or 5 nM concentrations of *fluvoxamine* resulted in a significant increase in mRNA expression levels of Notch1 and Hes1, in comparison to controls (Fig. 2A,B). In addition, assessment of proliferation marker Ki-67 demonstrated similar results to Notch1 and Hes1 at 1 nM and 5 nM, but not 0.1 nM, concentrations of *fluvoxamine*, in comparison to controls (Fig. 2C). However, fluvoxamine at 500 nM significantly decreased Notch1, Hes1 and Ki-67 mRNA levels, compared to controls (Fig. 2A–C, respectively).

**Figure 3:**
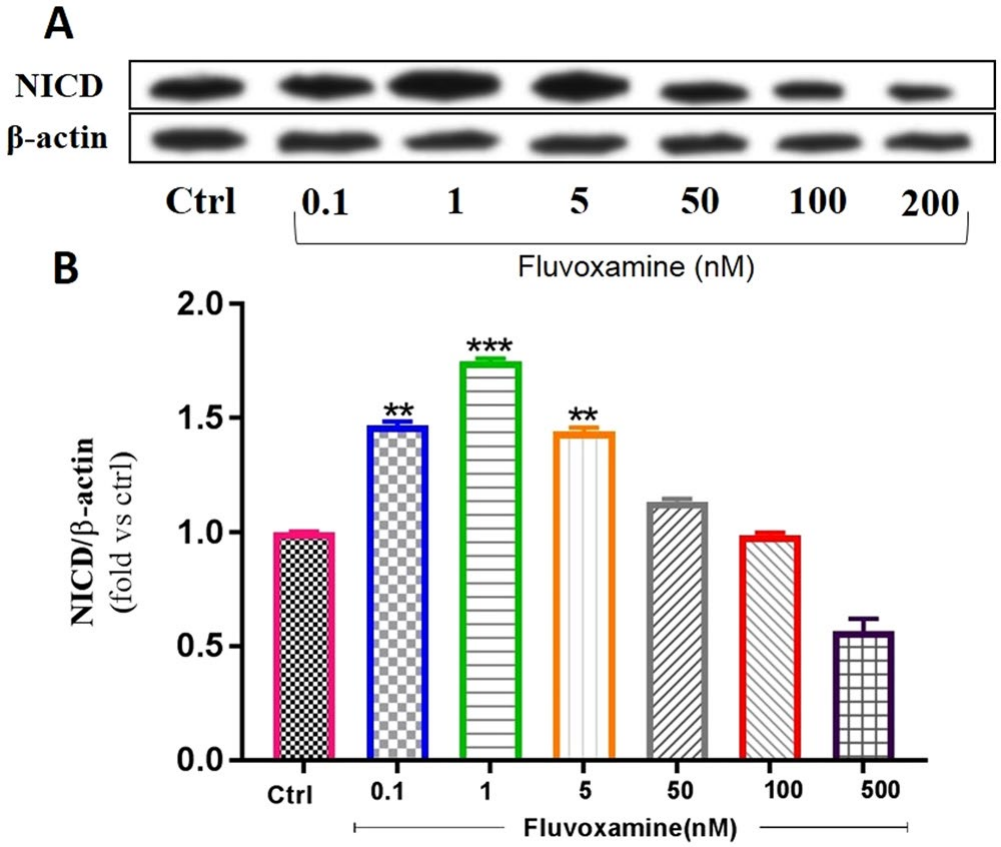
Effect of fluvoxamine on NICD protein expression levels. (**A**) Representative western blot showing NICD expressions. (**B**) Quantification of NICD expressions in all groups. β-actin was used as an internal control for normalization. Values are expressed as the Mean ± SEM. Each group included 5 replicates (n = 5). Statistical analyses were performed by one-way analysis of variance followed by Tukey’s test. Significance is indicated by *p < 0.05, **p < 0.01, ***p < 0.001 and ****p < 0.0001.

On the other hand, expression of Hes1 is regulated by Notch protein which is cleaved by γ-secretase releasing Notch intracellular domain (NICD). The latter moves into the nucleus and induces Hes1 expression that inhibits differentiation of NSCs^20^. Results showed that *fluvoxamine* at concentrations between 0.1 to 5 nM caused an increase in NICD protein expression in NSC cultures (Fig. 3A,B). Indeed, treatment with *fluvoxamine* at 0.1 or 5 nM induced ~1.5-fold increase in NICD levels, in comparison to controls (**p < 0.01), while 1 nM induced a maximal increase of ~1.75-fold (p < 0.001). Interestingly, *fluvoxamine* at higher concentrations suppressed NICD expression (**p < 0.01; Fig. 3B).

#### Fluvoxamine enhances neuronal differentiation of murine eNSCs

**Figure 4:**
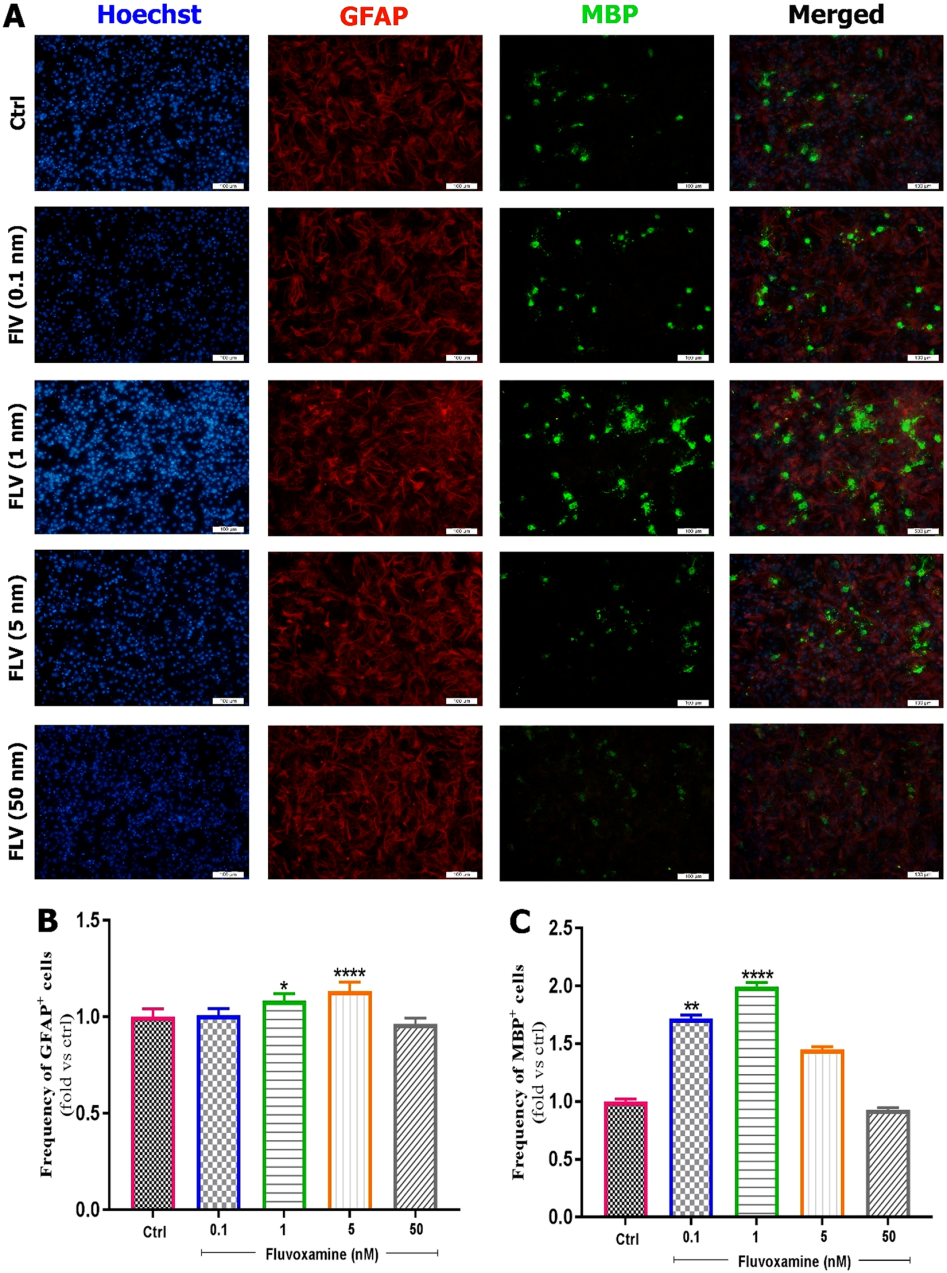
Effect of fluvoxamine on eNSC differentiation to glial and oligodendrocyte cells. (**A**) Representative immunofluorescence images of differentiated embryonic NSCs stained with GFAP (Glial Fibrillary Acidic Protein, an astrocyte marker, red) or MBP (Myelin Basic Protein, an oligodendrocyte marker, green) and Hoechst (a nuclei marker, blue) in control, and treated groups at day 6. White scale bars indicate 100 𝜇m. (**B**,**C**) Quantification of the frequency of GFAP and MBP positive cells in treated groups, normalized to the control group. Results showed that *fluvoxamine* at 1 and 5 nM concentrations are optimal for differentiation to MBP and GFAP positive cell, respectively. Each group included 3 replicates (n = 3). Values are presented as mean ± SEM for 15 field/well. Statistical analyses were performed by one-way analysis of variance followed by Tukey test. *p < 0.05; **p < 0.01; ***p < 0.001 and ****p < 0.0001.

**Figure 5:**
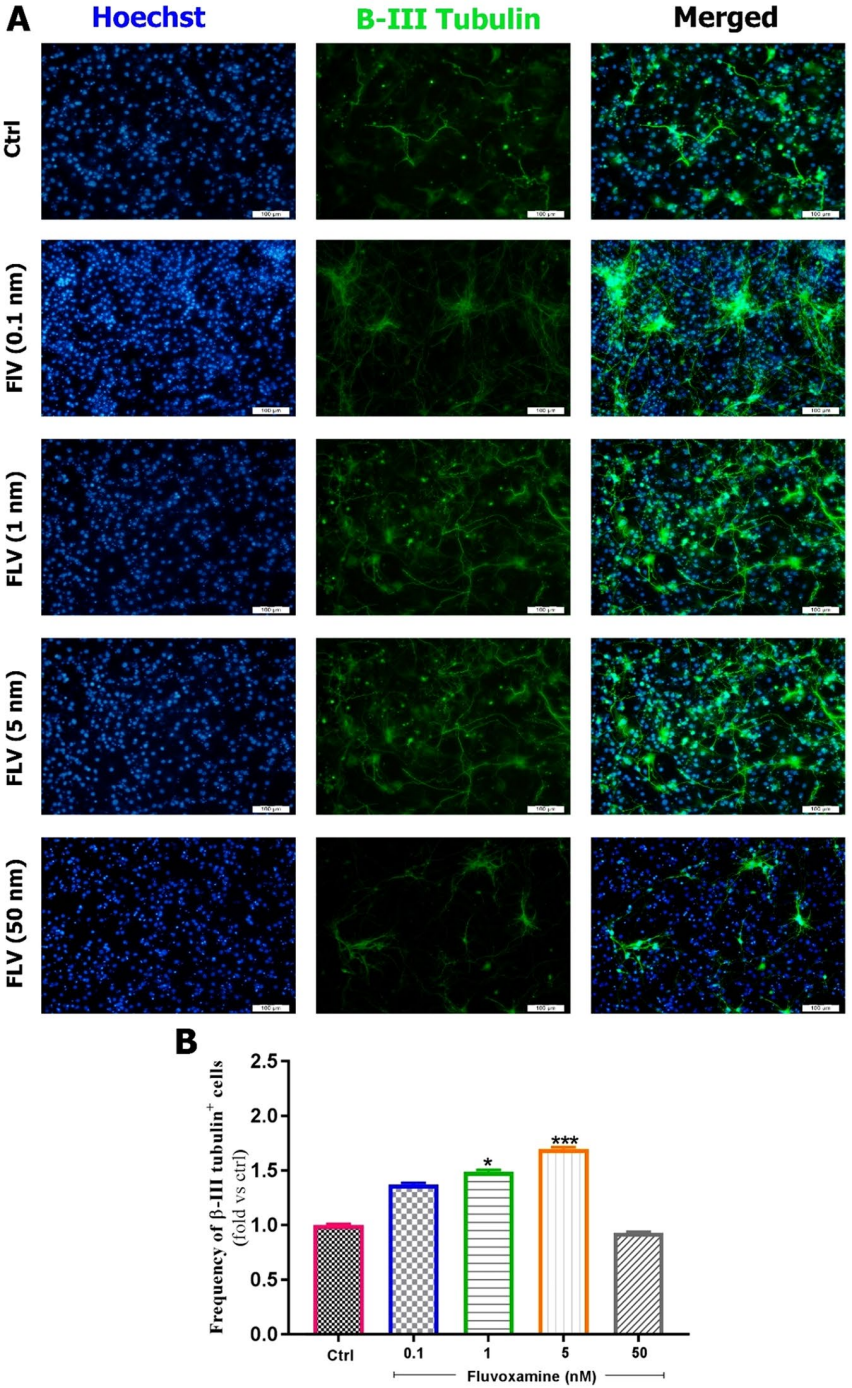
Effect of fluvoxamine on eNSC differentiation to neurons. (**A**) Representative images of differentiated embryonic NSCs stained with β-III Tubulin (a neuronal marker, green) and Hoechst (a nuclei marker, blue) in control and treated groups at day 6. White scale bars indicate 100 µm. (**B**) Quantification of the frequency of β-III Tubulin positive cells in treated groups, which has been normalized to controls. Results showed that 5 nM is the best concentration for differentiation of eNSCs to neurons. Each group included 3 replicates (n = 3). Values are reported as mean ± SEM for 15 field/well. Statistical analyses were performed by ANOVA followed by Tukey test. *p < 0.05; **p < 0.01; ***p < 0.001 and ****p < 0.0001.

Following treatment of eNSCs with various concentrations of *fluvoxamine* for 6 days, fluorescence images were captured. In this study, eNSC differentiation into GFAP-expressing astrocytes, MBP-expressing oligodendrocytes or β-III Tubulin-expressing neurons was tested by immuno-cytochemistry at 6 days after treatment. Results showed that eNSCs treated with 1 or 5 nM of *fluvoxamine* had a significant effect on the frequency of astrocytes (Fig. 4A). Indeed, the frequency of GFAP positive cells significantly increased in eNSCs treated with *fluvoxamine* at 1 nM (~1.08-fold; *p < 0.01) or 5 nM concentrations (~1.14-fold; ****p < 0.0001), in comparison to controls (Fig. 4B). In contrast, 0.1 nM or 1 nM concentrations of *fluvoxamine* showed a significant increase in differentiation of eNSCs toward oligodendrocytes (MBP positive cells), in comparison to controls (~1.77-fold; **p < 0.01 and ~2-fold; ****p < 0.0001; respectively) (Fig. 4A,C). Finally, *fluvoxamine* at 1 nM or 5 nM concentrations, but not 0.1 nM, showed a significant effect on neurons, in comparison to controls (Fig. 5A). Indeed, the frequency of β-III Tubulin positive cells increased significantly when murine cortical eNSCs were treated with 1 nM (~1.48-fold; *p < 0.05) or 5 nM of *fluvoxamine* (~1.7-fold; ***p < 0.001), in comparison to controls (Fig. 5B).

Taken together, *fluvoxamine* at an optimal concentration 5 nM enhanced the differentiation of eNSCs toward astrocytes and neurons, while 1 nM was the optimal concentration for differentiation toward oligodendrocytes.

### In vivo

#### The effect of fluvoxamine on clinical scores

**Figure 6:**
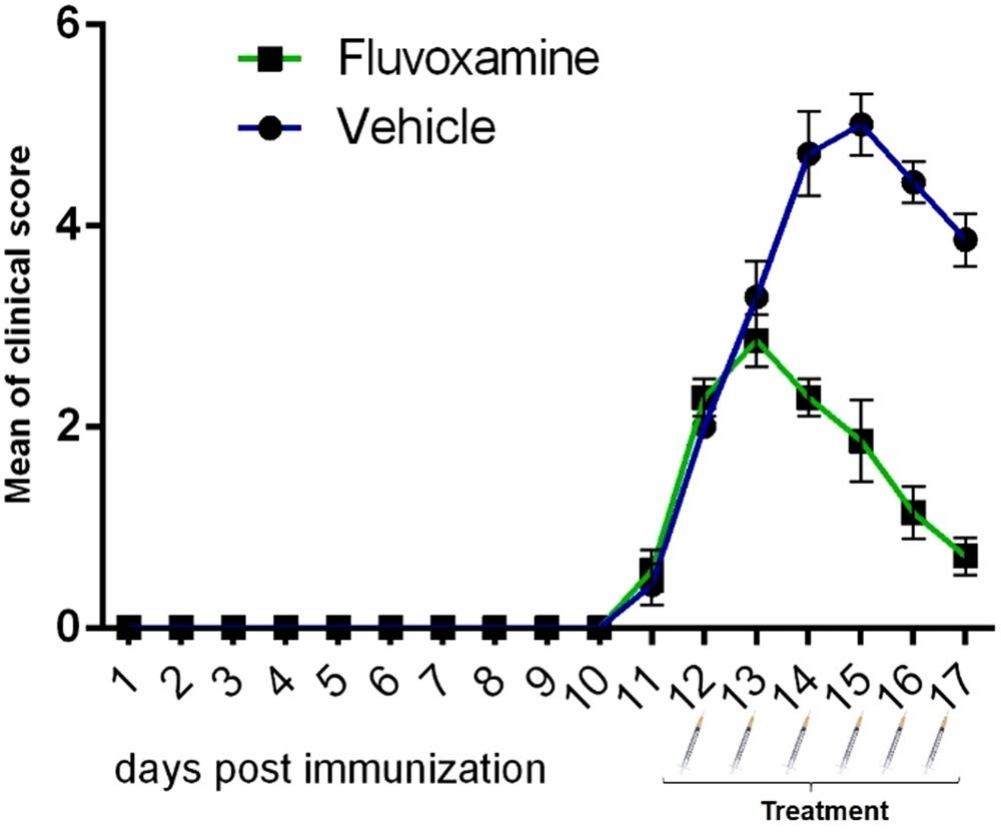
Administration of fluvoxamine ameliorated experimental autoimmune encephalomyelitis (EAE) clinical outcomes. EAE was induced in Lewis rats (day 0) which were treated at day 12 (scores > 2) with *fluvoxamine* (50 mg/kg) i.p. for 6 subsequent days. Daily clinical scores were measured from day 10 to day 17 in *fluvoxamine* and vehicle groups. Each group contained 7 rats. Data are expressed as mean ± SEM.

The peak clinical score of *fluvoxamine* treated rats was lower (1.85 at day 15), in comparison to PBS-treated rats (5 at day 15) (Fig. 6). At the end of study (day 17), these values declined to 0.71 in fluvoxamine treated rats and 3.84 in PBS treated rats.

#### The effect of fluvoxamine on pro- and anti-inflammatory cytokines

**Figure 7:**
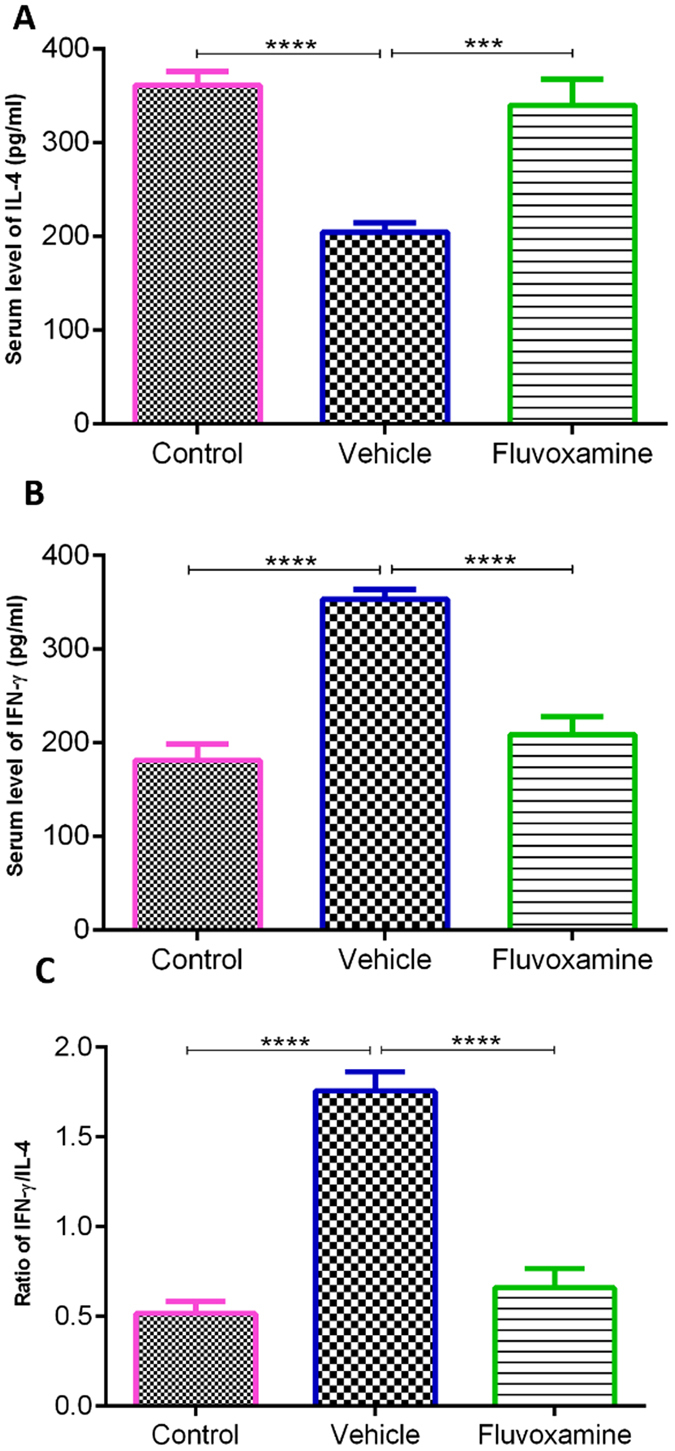
Concentrations of cytokines in serum samples using enzyme-linked immunosorbent assay. (**A**) IL-4 anti-inflammatory cytokine and (**B**) IFN-γ pro-inflammatory cytokine levels were measured in all rats on day 17. *Fluvoxamine* significantly reduced the levels of IL-4, while increasing the levels of IFN-γ, compared to EAE controls (p < 0.001 and p < 0.0001, respectively). (**G**) Ratio of IFN-γ/IL-4. Values represent the mean ± SEM. ***p < 0.001, ****p < 0.0001.

IFN-γ and IL-4 are the major cytokines that direct Th-1 and Th-2 development in EAE^21^. Results showed that serum levels of IL-4 in PBS-treated rats at day 17 were significantly lower (****p < 0.0001) than those of controls (204.3 ± 0.03 vs. 360.9 ± 14.5 pg/ml, Fig. 7A). However, treatment by *fluvoxamine* caused a significant increase (***p < 0.001) in IL-4 levels in EAE rats to 339.7 ± 27.8 pg/ml (Fig. 7A). On the other hand, IFN-γ levels in PBS-treated EAE rats were significantly higher (****p < 0.0001) than those of controls (353.0 ± 10.6 vs. 181 ± 2.0 pg/ml, Fig. 7B). In contrast, *fluvoxamine* treatment resulted in a significant decrease (****p < 0.0001) in serum IFN-γ, in comparison to PBS-treated EAE rats (208.6 ± 18.8 vs. 353.0 ± 10.6 pg/ml, Fig. 7B). The ratio of IFN-γ/IL-4, which acts as an indicator of Th-1/Th-2^21^, was found to be significantly higher in PBS-treated EAE rats, in comparison to controls (1.75 ± 0.10 vs. 0.51 ± 0.06; ***p < 0.001; Fig. 7C). However, this elevated ratio in PBS-treated EAE rats decreased significantly in *fluvoxamine* treated rats (0.65 ± 0.10; ***p < 0.001; Fig. 7C). These results confirmed that *fluvoxamine* ameliorates the severity of EAE by inhibiting IFN-γ release and promoting IL-4 production from Th1 and Th2 cells, respectively.

#### The effect of fluvoxamine on histopathological outcomes of EAE

**Figure 8:**
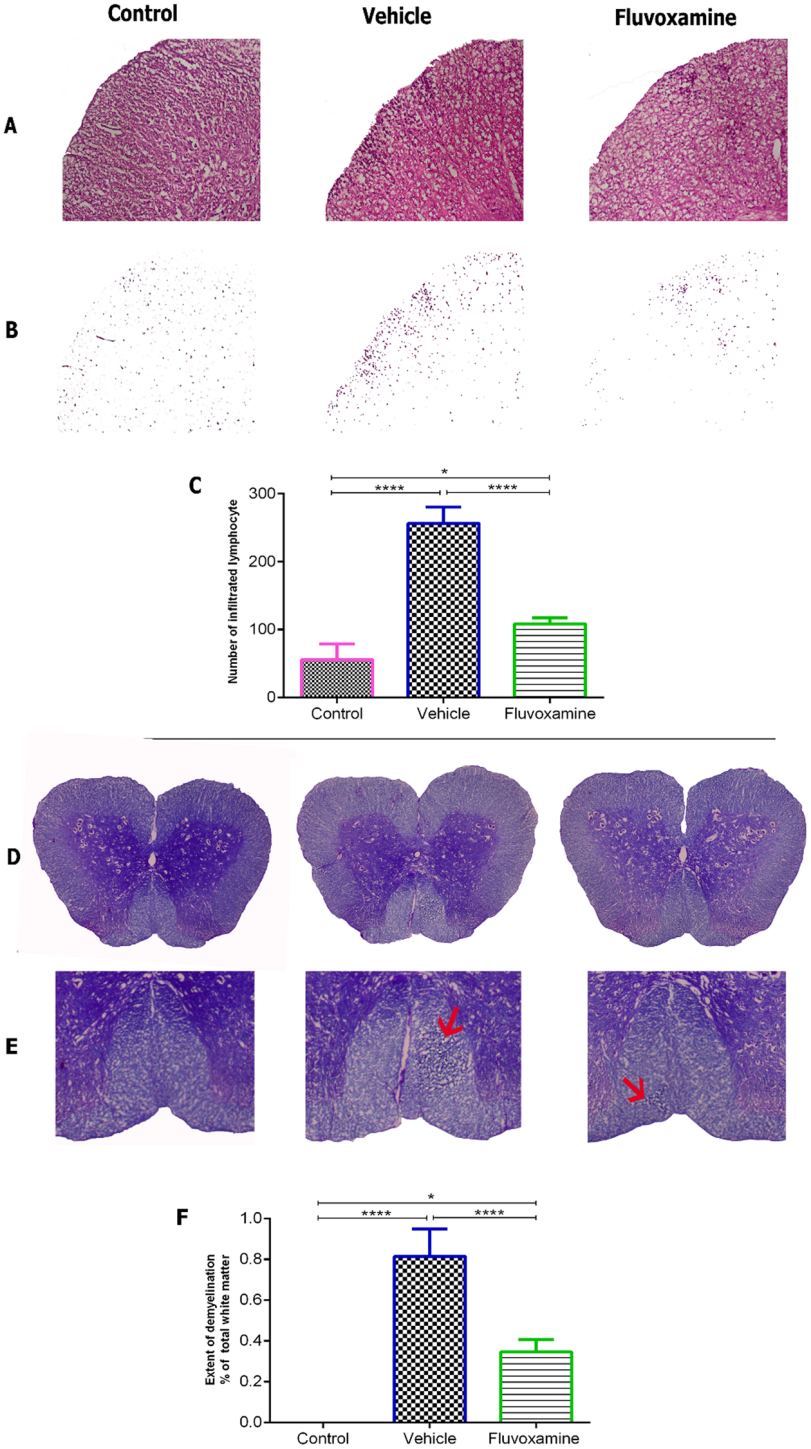
Fluvoxamine treatment enhanced histological outcomes in spinal cords of EAE rats. In order to detect inflammatory infiltration and demyelination, serial sections were analyzed by Hematoxylin and Eosin (**A**–**C**) and Luxol Fast Blue staining (**D**–**F**). (**A**) Inflammatory infiltration with extensive perivascular cuffing was limited and decreased in *fluvoxamine* treated EAE rats, in comparison to EAE control rats. (**B**) Infiltrated cells are shown without background tissue, a modification from panel (A). (**C**) Infiltration of immune cells was significantly reduced in fluvoxamine group, compared to vehicle (****p < 0.0001). (**D**,**E**) Red arrows indicates demyelination area which is reduced in *fluvoxamine* group, compared to EAE vehicle rats. (**F**) Quantitative analysis for percent of demyelination area of total white matter using Luxol Fast Blue staining. *Fluvoxamine* induced a reduction in demyelination area (0.81%), compared to vehicle (0.34%). Data are expressed as mean ± SEM from seven rats per group.

To confirm whether *fluvoxamine* treatment could provide protective effects on the pathological changes in the CNS, neuropathological analysis was conducted on lumbar spinal cords at day 17 post immunization. Blind qualitative analysis indicated a diffuse infiltration of lymphocytes into CNS white matter in the lumbar spinal cords of EAE rats, which was decreased in *fluvoxamine* treated EAE rats (Fig. 8A,B). In addition, quantitative analysis showed that the mean number of infiltrated cells/field in *fluvoxamine* treated EAE rats (108.4 ± 9.1) was significantly lower (****p < 0.0001) in comparison to PBS-treated EAE rats (256.4 ± 24.1) (Fig. 8C). Similarly, surface areas of demyelination plaques at day 17 were markedly reduced (****p < 0.0001) in *fluvoxamine* treated EAE rats (0.34% ± 0.06), in comparison to PBS-treated EAE rats (0.81% ± 0.13) (Fig. 8D–F).

#### Assessment of oligodendrocytes and astrocytes

**Figure 9:**
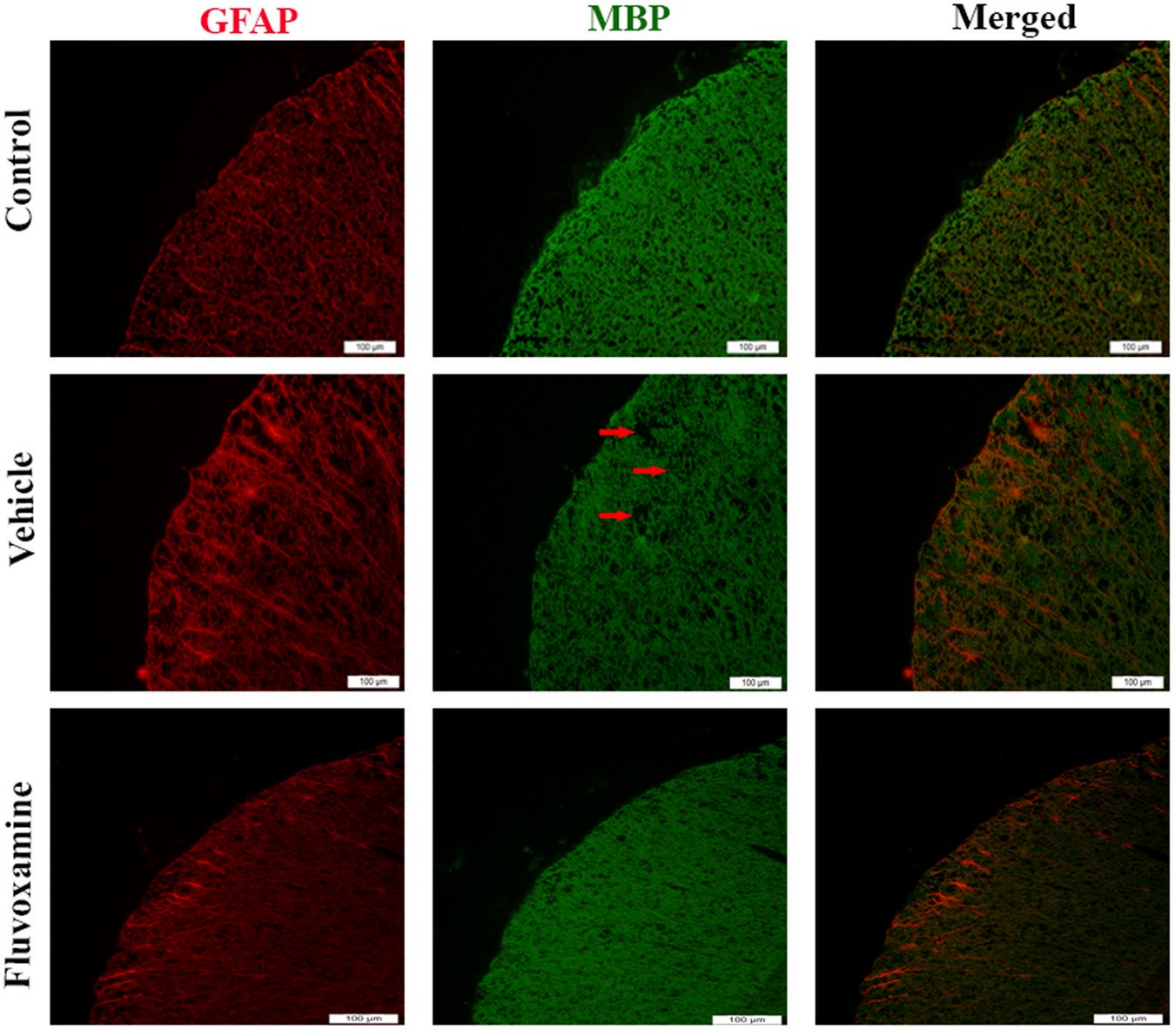
Immunohistochemical staining of myelin basic protein (MBP) and glial fibrillary acid protein (GFAP). *Fluvoxamine* caused an increase in MBP in demyelination area (red arrow) and GFAP positive cells (red). Scale bars = 100 μm.

Immunofluorescent analysis of lumbar spinal cords clearly showed increased demyelinated plaques, as demonstrated by MBP deficient areas, and activation of GFAP-expressing cells in control untreated EAE rats (Fig. 9). However, *fluvoxamine* treated EAE rats showed a reduction in demyelinated areas, demonstrated by an increase in MBP in demyelination areas, compared to controls. Also, *fluvoxamine* therapy revealed intense GFAP positive staining, in comparison to controls (Fig. 9).

**Figure 10:**
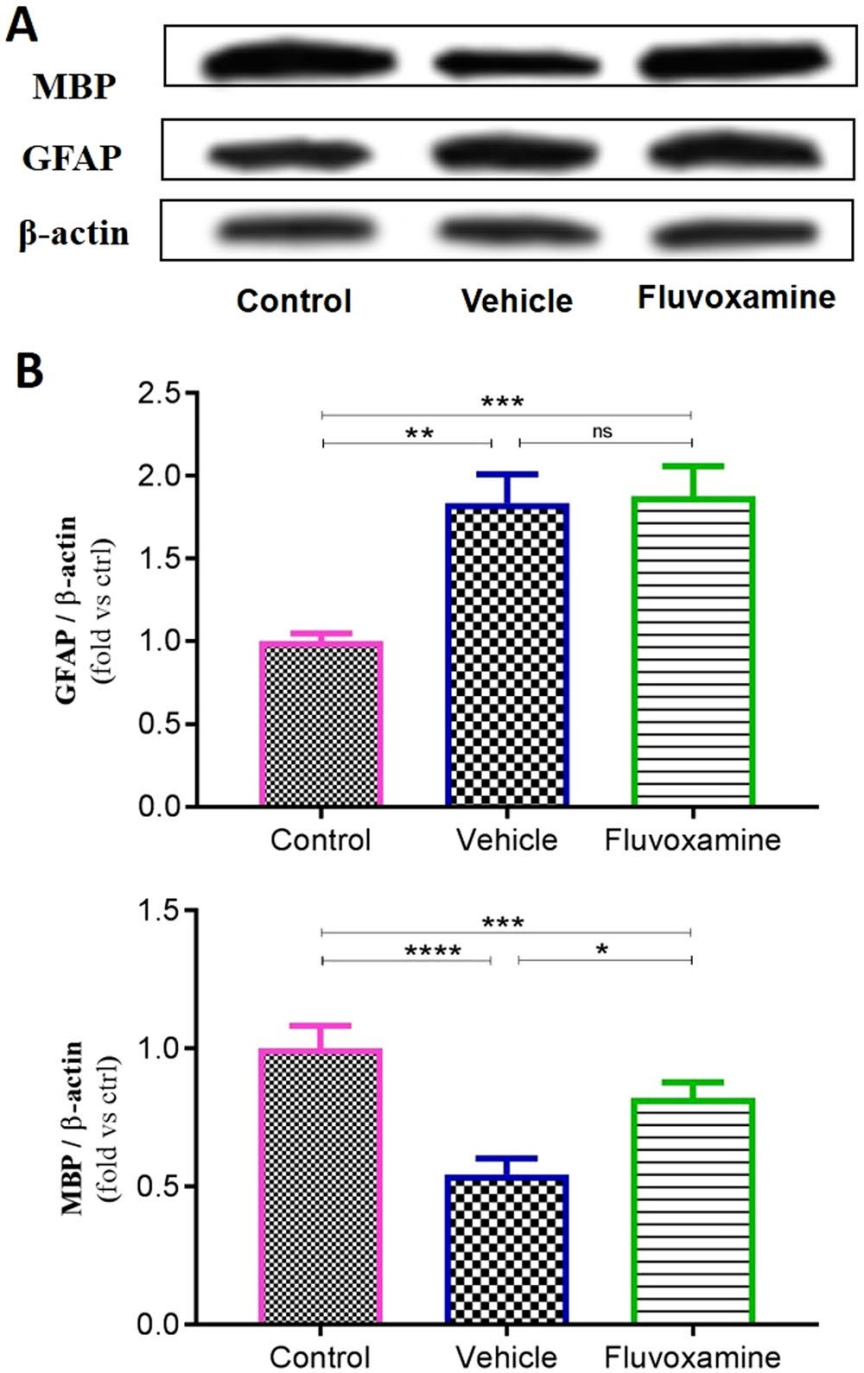
Western blot assessment of glial fibrillary acid protein (GFAP) and myelin basic protein (MBP). (**A**) Representative western blot of GFAP and MBP protein expression in the spinal cord. β actin was used as housekeeping control. (**B**) Quantitative analysis was performed using LabWorks Software. Values are expressed as the Mean ± SEM. Each group included 7 rats (n = 7). Statistical analyses were performed by one-way analysis of variance followed by Tukey’s test. Significance is indicated by *p < 0.05, **p < 0.01, ***p < 0.001 and ****p < 0.0001.

Using western blotting, *fluvoxamine* treated or untreated EAE rats showed a similar significant increase (~1.83-fold) in GFAP protein expression levels in comparison to control rats (Fig. 10). On the other hand, protein expression levels of MBP significantly decreased in untreated EAE rats (~0.54-fold; ****p < 0.0001) in comparison to controls, while this reduction in *fluvoxamine* treated rats reached ~0.82-fold (***p < 0.001), in comparison to controls (Fig. 10).

#### The effect of fluvoxamine on serum lactate as an EAE progression biomarker

**Figure 11:**
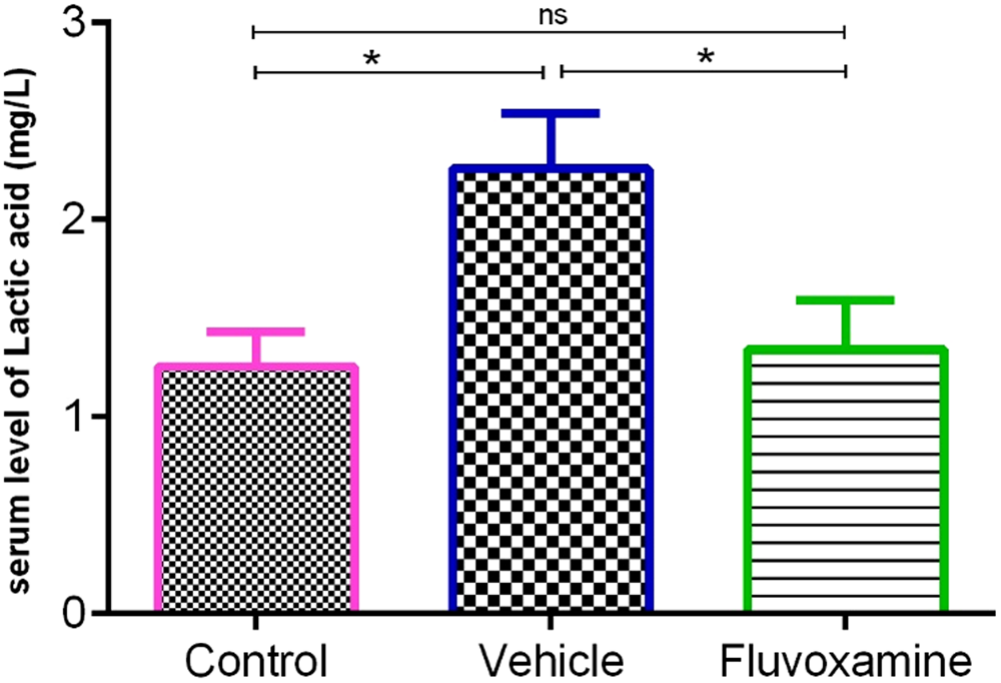
Measurement of lactate levels in serum samples using HPLC. Induction of EAE caused an increase in lactate concentrations, however, administration of *fluvoxamine* reverted the serum levels of lactate back to normal; Data are expressed as Mean ± SEM.

Since circulating lactate is a potential biomarker of MS and EAE progression^22–24^, its concentration was measured in the serum of all experimental groups (Fig. 11). Results showed that *fluvoxamine* caused a significant decrease (*p < 0.05) in the lactate levels (1.34 ± 0.24 mg/l), in comparison to PBS-treaded EAE rats (2.26 ± 0.27). Indeed, *fluvoxamine* induced decrease in the lactate levels was comparable to their original control values (1.25 ± 0.17 mg/l) (Fig. 11).

## Discussion

It has been reported that cells within the mammalian subventricular zone (SVZ) could be activated in MS patients to promote gliogenesis and hence could be used for cell therapy in pathological demyelination situations^25, 26^. Therefore, we investigated the effect of nanomolar concentrations of fluvoxamine on NSCs originating from the rat SVZ region which we demonstrated to have a beneficial role by increasing cell viability and neurospheres counts. Since the effect of *fluvoxamine* on NSCs has never been studied before, we compared our results to other SSRI drugs, mainly *fluoxetine*. In accordance with our data, *fluoxetine* was reported to have a protective role against cell death in concentrations between 100 pM to 1 µM and a dose-dependent effect on the proliferation of NSCs, isolated from cerebral cortices of mice^27^. It’s important to note that the optimal concentration for proliferation of NSCs using *fluoxetine* was higher than 10 nM whereas only 1 nM was enough for *fluvoxamine* to exert its effects in our study. In contrast, it has been shown that the number of neurospheres decreased following *fluoxetine* treatment at concentrations ranging between 0.1 to 1 µM, using NSCs from SVZ region of adult mice^28^. Moreover, the adult hippocampus of *fluoxetine* treated rats (5 mg/kg) showed increased BrdU-positive cells, a marker of newly born cells^29^.

At the molecular level, several signaling pathways have been implicated in the mechanisms of proliferation and differentiation of NSCs and in regulating CNS development, among which Notch1 signaling pathway^30^. For instance, it has been recognized that Notch1 and Hes1 could promote cell proliferation and self-renewal of NSCs^31, 32^. It has been confirmed that upon activation of Notch, the NICD is released from the membrane and translocates to the nucleus to induce the expression of transcriptional repressor genes such as Hes1 which inhibits neuronal differentiation. Thus, activation of Notch signaling leads to maintenance of the neural stem cell population^33, 34^.

In accordance, expression of Ki-67, as an endogenous marker for cell proliferation, is tightly associated with active cell proliferation^35, 36^. In this study and in agreement with cell viability and neurospheres results, mRNA expression levels of Notch1, Hes1 and Ki-67 but also protein levels of NICD showed a similar trend in enhancing NSCs proliferation when low concentrations of *fluvoxamine* were used. The concentrations of 1 nM and 5 nM used here are considered physiologically low. Based on the above, our data indicate that *fluvoxamine* acts through Notch1 signaling pathway in order to enhance cell proliferation.

In this study and for the first time, our *in vitro* data demonstrated an upregulation of gliogensis in response to administration of low nanomlolar concentrations of *fluvoxamine*. Indeed, fluvoxamine caused a significant increase in the differentiation of eNSCs into GFAP-expressing astrocytes, MBP-expressing oligodendrocytes and β-III Tubulin-expressing neurons. In accordance, similar results were obtained in rat hippocampus following *fluoxetine* (5 mg/kg) treatment where ~75 percent of newborn cells were neurons^29^. Similarly, *fluoxetine* at nanomolar concentrations was shown to increase the number of β-III tubulin (Tuj 1)-positive cells while it was ineffective on NSC differentiation toward GFAP-positive cells, in contrast to our results^27^. Finally, while other reports focused on proliferation or neurogenesis activities of SSRIs drugs, mainly *fluoxetine*, our study covered the proliferation and differentiation toward astrocytes, oligodendrocytes, and neurons. Indeed, we demonstrated the high ability of *fluvoxamine* at physiologically low concentrations of 5 nM to stimulate differentiation into astrocytes and neurons, while even lower concentrations of 1 nM dramatically promoted differentiation toward oligodendrocytes.

There are no clear guidelines in the literature about choosing the best antidepressant drug for MS patients. Despite the wide use of tricyclic antidepressants, a placebo-controlled trial reported modest beneficial effects of *desipramine*, with considerable side effects in one-half of the patients^7^. On the other hand, although investigations on selective serotonin reuptake inhibitors (SSRI) are rare, a previous study indicated MS exacerbation following *fluoxetine* administration^37^ while a more recent report demonstrated some efficacy for the use of *sertraline* ^38^. *Fluvoxamine*, another member of the SSRI family, has been rarely investigated whereas most of the studies have used *fluoxetine* on inflammatory diseases and MS. Importantly, while *fluoxetine* was shown to cause a reduction in dopamine synthesis^39^, *fluvoxamine* did not have any effect^40^. However, recent studies clearly reported a beneficial role of *dopamine* in EAE recovery^41, 42^. Since limited studies have evaluated the effects of SSRIs drugs on oligodendrogenesis, *fluvoxamine* was used in this study in order to evaluate its anti-inflammatory effects as wells as its involvement in the re-myelination process.

Our results on EAE *in vivo* model demonstrated that administration of *fluvoxamine* (50 mg/kg) leads to lower clinical scores and EAE amelioration by attenuating immune cell infiltration into the CNS and reducing plaques demyelination. Due to the lack of relevant studies on *fluvoxamine*, our findings were compared with those performed on SSRI drugs, mainly *fluoxetine*. Indeed, it was reported for the first time in 1990 that *fluoxetine* had adverse effects on MS patients^37^. Moreover, a study on the anti-inflammatory effect of *fluoxetine*, showed a reduction in the levels of CNS inflammation in *fluoxetine* treated EAE rats^11^. Furthermore, *fluoxetine* was demonstrated to act as a potent anti-inflammatory agent at various concentrations ranging from 10 to 60 mg/kg, as measured by comparing paw edema inflammation in the rat. Indeed, the latter study showed that *fluoxetine* had a higher anti-inflammatory activity, compared to either *imipramine*, a tricyclic antidepressant drug, or *trazodone*, a serotonin antagonist and reuptake inhibitor^43^.

On the other hand, previous studies confirmed that Th1 cells secrete TNF-α and IFN-γ which activate phagocytic macrophages leading to the destruction of myelin and oligodendrocytes^44, 45^. In accordance, our results showed a correlation between lower IFN-γ levels along with attenuated areas of demyelination plaques. Indeed, our study further confirmed that *fluvoxamine* ameliorated the severity of EAE rats by reducing pro-inflammatory IFN-γ release and enhancing anti-inflammatory IL-4 cytokine production levels from Th1 and Th2 cells, respectively. On the other hand, it appears that the sharp increase in IL-4 reported in this study is responsible for the inhibition of Th1 cells and preventing an increase in IFN-γ secretion. In agreement, IL-4 was previously shown to inhibit the release of IFN-γ and that Th2 cells are sensitive to IFN-γ, which can strongly inhibit its production^46^.

GFAP-expressing cells respond to injuries through a process commonly referred to as reactive astrogliosis, which is an abnormal increase in the number of astrocytes due to the destruction of nearby neurons from CNS infection, trauma, stroke, ischemia, autoimmune responses, and neurodegenerative disease^47–50^. Despite the various studies, the best-known aspect of reactive astrogliosis is still incomplete. Several specific molecular and morphological features have been observed in astrocytes during reactive astrogliosis in both human pathology and animal models, mainly upregulation of hallmark of GFAP positive cells^51–53^. After injury and disease, astrocytes become reactive and prevent regeneration^54^, in line, we found an activation of GFAP-expressing cells in spinal cord of EAE rat. In accordance, this upregulation in astrocyte activation, concomitant with elevated level of IFN-γ, which could explain the greater demyelinated plaque in untreated EAE rat, compared to normal. On the other hand, *fluvoxamine* treatments seem to modulates immune system function to inhibit pathological condition as confirmed by decline in level of IFN-γ and attenuated level of spinal cord inflammation and demyelination. Previous studies have also been suggested that astrocytes can become activated and promote regeneration^54–57^.

The most plausible mechanism for the observed recovery in EAE severity and remyelination following *fluvoxamine* treatment seems to involve reduced levels of IFN-γ. Interestingly, an important study by Wensky and colleagues in 2005 demonstrated that IFN-γ determines distinct clinical outcomes in autoimmune encephalomyelitis models^58^. Similarly, Butovsky *et al*. reported that low doses of IFN-γ had no effect on insulin growth factor-1 (IGF-1) gene expression, whereas high doses of IFN-γ lead to inhibition of oligodendrogensis, through induction of TNF-alpha and eventually inhibition of IGF-1^59^. The latter is involved in the recruitment of oligodendrocyte progenitor cells (OPCs) following demyelination, delays EAE onset and improves clinical outcome^60^. In addition, IGF-I plays a vital beneficial role in neural tissue survival and renewal^59^ since its expression is increased in the lesion area during restoration and proliferation of OPC, which confirms IGF-1 role in the remyelination process^61^. In accordance, IGF-1 knock-out mice were shown to have a drastic reduction in the number of OPC and mature oligodendrocytes^62, 63^. Moreover, several studies have clearly demonstrated that astrocytes stimulated by IFN-γ affected MBP and displayed myelin peptides in an MHC class II restricted manner to T cells, leading to the activation of encephalitogenic T cells^64–66^. As previously shown, it is likely that high concentrations of IFN-γ results in the inhibition of IGF-1 production by glial cells, considered as a harmful path^59^, and thus myelin repair would be attenuated. In line, our results showed increased astrocytes (GFAP+ cells) in *fluvoxamine* treated rats, which was concomitant with lower levels of IFN-γ, similar to normal rats, a plausible mechanism for a limited demyelinated area in *fluvoxamine* treated EAE rats.

In accordance, it has been reported in lipopolysaccharide (LPS)-stimulated microglial cells that increased levels of TNF-α and IL-6 pro-inflammatory cytokines were further suppressed by *fluoxetine* treatment, suggesting that its therapeutic effect could be mediated by modulating microglial activation as seems to be involved in *fluvoxamine* treated EAE model^67^. In the current study, we demonstrated that the extent of demyelinated area was decreased in *fluvoxamine*-treated EAE rats, in comparison to EAE controls, whereas the number of mature oligodendrocytes and astrocytes increased.

Given the rapid rate of improvement in rats with acute EAE, it seems likely that the observed limited demyelination and enhanced oligodendrogenesis in this *fluvoxamine* treated model can be attributed in part to activation of astrocytes by decreased IFN-γ, but it could also be ascribed to growth factors such as IGF-I which are produced in the demyelination area. Further investigation would be needed in order to elucidate the exact mechanism by which IFN-γ and IGF-1 are exerting their effects.

In view of biomarker assessment of MS or EAE progression, we demonstrated a reduction of lactate in *fluvoxamine* treated EAE rats, compared to untreated EAE rats, which further confirms the beneficial role of *fluvoxamine* in treatment of EAE, using lactate as a biomarker for response to therapy. A direct correlation between MS severity and serum lactate, the most important monocarboxylate in the glycolysis pathway, has been previously reported^22, 68–70^. Moreover, we demonstrated that the reduction of lactate levels in *fluvoxamine* treated EAE rats was concomitant with limited demyelination area. Therefore, we suggest that lactate has been consumed by *fluvoxamine* treated rats during remyelination process to make energy and fatty acids. Indeed, it has been shown that pyruvate and lactate as main substrates within the CNS can be used to make ATP and fatty acids, required to synthesize myelin during myelination^71–75^.

In summary, our study demonstrated that *fluvoxamine*, in addition to its confirmed role in mood disorder therapy, could serve as a candidate clinical treatment for attenuating neuroinflammation and stimulating oligodendrogenesis in neurological diseases, particularly MS patients.

## Materials and Methods

### In Vitro

#### Experimental animals

Adult Lewis rats (8–12 weeks old, 150–175 g) were purchased from DarouPakhsh institute (Karaj, Iran). Embryonic day 14 (E14) Lewis rat embryos were used to generate neural stem cells (NSCs). The Institutional Animal Care and Use Committee (IACUC) of Yasuj University of Medical Science approved all experimental procedures and animal use in this study. Rats were maintained and housed under pathogen-free conditions with constant temperature and humidity control at the Animal Breeding Center^76^. Surgical procedures were performed under deep anesthesia. Housing of the animals and all animal experimental procedures were carried out in accordance with the guidelines of the Iranian Agriculture Ministry, which conforms to the provisions of the Declaration of Helsinki (as revised in Brazil in 2013), and of the European Communities Council Directive (86/609/EEC). All efforts were made to reduce the number of animals used and their suffering.

#### Isolation and expansion of NSCs

Primary cultures of embryonic NSCs were performed by the neurosphere method, as described previously^77^. Briefly, pregnant rats (day 14) were sacrificed after anesthesia and the harvested embryos transferred to sterile tubes containing cold PBS. The cortices were then mechanically disrupted into single cells by repeated pipetting in a serum-free neurosphere medium called N2 medium. This medium consists of DMEM/F12 (1:1), 0.6% (w/v) glucose, 0.1125% (w/v) sodium bicarbonate, 2 mM L-glutamine, 5 mM HEPES, 100 µg/mL human apotransferrin, 20 nM progesterone, 30 nM sodium selenite, 60 µM putrescine, and 25 µg/mL insulin. Cells were then plated in T25 flasks in suspension at a density of 1 × 10^5^ cells/mL in proliferation medium consisting of the above N2 medium supplemented with 20 ng/mL basic fibroblast growth factor (bFGF; R&D Systems, Inc., Minneapolis, MN, USA) and 2 mg/mL heparin (Sigma-Aldrich). Cells were maintained in an incubator with a humidified atmosphere containing 5% CO_2_ at 37 °C for 5–6 days. Neurospheres were then harvested by centrifugation, dissociated using trypsin and EDTA (Sigma-Aldrich), and reseeded for the following experiments.

#### Cell viability assay

Cell viability of NSCs was assessed by employing the 3-(4, 5-dimethylthiazol-2-yl)-2, 5-diphenyltetrazolium bromide (MTT) colorimetric assay, based on the amount of insoluble purple formazan product. Briefly, dissociated neurosphere derived cells from primary cultures were seeded at a density of 5000 cells in 96-well plates and treated with different concentrations of *fluvoxamine* (0.1, 1, 5, 50, 100 and 500 nM) for 48 hours and cultured in a humidified atmosphere of 5% CO_2_ at 37 °C. Due to lack of similar study, the concentrations of *fluvoxamine* chose according to other similar studies, which have used *fluoxetine* with range between 0.1 nM and 50 µM^14, 27, 66^. After the treatment, *fluvoxamine*-containing medium was removed, wells were gently washed twice with PBS, and then 200 μl of 0.5 mg/ml MTT in PBS was added to each well. The plate was incubated at 37 °C for 4 h. Cells were then disrupted in solubilizing solution (1:1 ratio of dimethyl sulfoxide and ethanol). The formazan dye produced by viable cells was quantified in an ELISA microplate reader at an absorbance of 460 nm. A total of five independent experiments were conducted.

#### Determination of neurosphere frequency using Neurosphere assay

Dissociated neurosphere derived cells were seeded at a density of 5000 cells in 96-well plates and treated with different concentrations of *fluvoxamine* (0.1, 1, 5, 50, 100, and 500 nM) (n = 15 replicates for each concentration). After 5 days, culture plates were monitored using an Olympus inverted light microscope. The total number of neurospheres with a diameter of >50 µm were counted and expressed as the percent neurosphere-forming frequency per well with respect to the total number of cells that were plated initially. The mean of all wells (n = 15) was considered as the sphere forming frequency for each condition.

#### Real-Time PCR

The NSCs were cultured and allowed to proliferate for 5 days in proliferation medium in the presence of different doses of *fluvoxamine*. Total RNA from NSCs was extracted using QIAGEN RNeasy Kit (Qiagen, Tokyo, Japan), and then cDNA was synthesized with the High-Capacity cDNA Reverse Transcription kit using random primers (Applied Biosystems, USA). Quantitative real-time PCR (qPCR) was performed by the StepOne Real-Time PCR system (Applied Biosystems, Inc, Foster City, CA, USA). Real-time PCR was carried out with RealQ Plus 2x Master Mix Green (Ampliqon, Denmark) according to manufacturer’s instructions. The primer sequences used were the following: Hes 1, F: TTCCTCCCATTGGCTGAAAG and R: CCAGCTCCAGATCCAGTGTGAT; Notch1, F: TGGTTCAGGGCGGTGCTCA and R: CAGACACCTGCTTCCCAAAAGG; Ki-67, F: GAGCAGTTACAGGGAACCGAAG and R: CCTACTTTGGGTGAAGAGGCTG; GAPDH, F: ATCTTCTTGTGCAGTGCCAGC and R: CCTTGACTGTGCCGTTGAACT. The specificity of PCR products was confirmed by melting curve analysis (data not shown). The PCR conditions were as follows: initial activation at 95 °C for 15 min, then 35 amplification cycles consisting of denaturation at 95 °C for 15 s, annealing at 57 °C for 30 s, and extension at 72 °C for 30 s. The relative changes in gene expression levels were determined by the Comparative CT (ΔΔCT) method. All reactions were performed in triplicate using GAPDH as an internal control.

#### Western blotting

NSCs were cultured and allowed to proliferate for 5 days in proliferation medium in the presence of different doses of *fluvoxamine*. Cells were then homogenized on ice and lysed in a lysis buffer containing 50 mM Tris–HCl (pH 7.5), 150 mM NaCl, 0.5% deoxycholic acid, 1% Nonidet P40, 0.1% SDS, 1 mM PMSF, and 100 mg/ml leupeptin. Protein content was measured using a Bio-Rad colorimetric protein assay kit (Bio-Rad, Hercules, CA, USA). An equal amount of total protein (40 μg) was resolved on 8–15% sodium dodecyl sulfate polyacrylamide gel and then transferred onto a nitrocellulose membrane. The membranes were blocked for 1 h in 5% skim milk solution, and then incubated with primary antibody against NICD (1:500, Abcam, USA) or β-actin (1:1000, Santa Cruz Biotechnology Inc. CA, USA) for an overnight on shaker at 4 °C. After washing, horseradish peroxidase-conjugated species appropriate secondary antibodies were applied. Incubations were performed at room temperature. Immunoreactive proteins were detected with an enhanced chemiluminescence Western blotting detection system. The relative density of the protein bands was scanned by densitometry using MyImage (SLB, Seoul, Korea), and quantified by image analysis software for gel documentation (LabWorks Software Version 3.0, UVP Inc., CA, USA). When spinal cords were used, a similar protocol was performed with primary antibodies against MBP (1:1000, Abcam, USA), GFAP and β-actin (1:1000, Santa Cruz Biotechnology Inc. CA, USA).

#### Neural stem cells differentiation

For differentiation, neurospheres (passage 2) were mechanically dissociated into single cells using 0.05% trypsin-EDTA (Gibco, USA), counted and the resulting cells seeded in poly-L-ornithine (15 mg/mL) coated 12-well plates (Sigma-Aldrich) at a density of 1 × 10^6^ cells/well containing N2 medium without bFGF and EGF. In the experimental groups, cells were treated with 0.1, 1, 10 or 50 nm concentrations of *fluvoxamine*, dissolved in N2 medium containing 1% free bovine serum albumin (BSA, Gibco, USA) at a final concentration of 0.01%. The oligodendrocyte promoting factor PDGF (30 ng/ml) was used as a positive control. The culture medium was changed every other day for a total of 5 days.

#### Immunofluorescence (IF) analysis

After 5 days, differentiated cultures were used for IF analysis. Cells in 12-well plates were fixed in 2% paraformaldehyde (PFA) for 20 min, washed with PBS, and permeabilized using 20% tween for 20 min, and then blocked in PBS, 2% triton, 5% horse serum (PBSTS) for 20 min. Cells were then incubated overnight with GFAP and MBP primary antibodies (Sigma Aldrich, USA) at 1:800 and 1:500 dilutions, respectively^78^. Wells were then washed in PBS and incubated for 1 hour with Alexa Fluor 488 or 568 (1:500 dilution) conjugated anti-mouse or anti-rabbit secondary antibodies, respectively. Representative pictures of each well (10–12 fields/well) were taken using a fluorescent microscope (Olympus IX-71) equipped with a Canon EOS digital camera. Cell counts were then performed and data presented as percent positive cells per treatment condition.

### In vivo

#### Experimental Animals

Adult female Lewis rats (8–12 weeks old, 150–175 g) were purchased from DarouPakhsh institute (Karaj, Iran). All animals were kept in standard conditions with unlimited access to food and water. IACUC, housing and surgical procedures were performed as mentioned in the previous section.

#### Experimental autoimmune encephalomyelitis (EAE) induction

Rats were anesthetized with isoflurane (Abbott Labs, USA) and then injected subcutaneously over the flank with 200 μl of a 1:1 (vol/vol) mixture of 1 g of Guinea Pig Spinal Cord (GPSC) in 1 ml PBS and Complete Freund’s Adjuvant (CFA, Sigma Aldrich) and 1 mg/ml enriched Mycobacterium tuberculosis bacteria.

#### Clinical evaluation

Rats were evaluated and scored for clinical signs of the disease by at least 2 investigators from day 7 to day 17 post immunization using a 0–8 point scale^79^, as follows: 0 = normal; 1 = flaccid/limp tail; 2 = hind limb weakness causing righting difficulty from a supine position; 3 = hind limb weakness causing righting inability ≥8 sec from a supine position; 4 = hind limb weakness causing limping and abnormal gait; 5 = partial (one limb hind limb paralysis or extensive hind limb weakness such that the hind limbs cannot contribute to mobility; 6 = total (both) hind limb paralysis plus forelimb weakness; 7 = hind limb paralysis and forelimb weakness or paralysis resulting in a side resting position; 8 = moribund requiring sacrifice or inadvertent death.

#### Treatment of animals

Rats were randomly divided into 3 groups of: (A) Control PBS-treated rats (n = 7); (B) Vehicle PBS-treated EAE rats (n = 7) and (C) *fluvoxamine* (50 mg/kg) treated EAE rats (n = 7). All administrations were done intra-peritoneally (i.p.). In order to have a clinically relevant protocol, treatment was given intraperitoneally (*i*.*p*.) for 6 consecutive days (from day 12 to 17), starting on the day of clinical symptom onset (score ≥ 3), until sacrifice.

Prior to treatment, animals were weighed and the volume of *fluvoxamine* to be injected (1 ml/250 g of body weight) was calculated accordingly^80^. *Fluvoxamine* was freshly prepared by dissolving it in PBS then injected intraperitoneally (*i*.*p*.) at a dose of 50 mg/kg, which was chosen based on a previous study as the most effective dose in reducing inflammation^81^.

#### Histopathological analysis

After 17 days’ post immunization, rats were deeply anesthetized with ketamine/xylazine (5/1) and then perfused via the left ventricle with 30 ml PBS (0.1 M). Since the most significant EAE histopathological changes were detected in the lumbar region of the spinal cord, it was removed and immersed in 4% PFA for 24 h. Fixed tissues were then paraffin-embedded, and 6 μm sections were prepared from the lumbar spinal cords. Sections were then de-paraffinated and hydrated through xylol and alcohol with a routine protocol. A total of 10 systematic randomly selected sections of lumbar spinal cord from each animal were stained with luxol fast blue (LFB) and hematoxylin and eosin (H&E) for measuring the extent of demyelination area and infiltration state, respectively. Sections were then scanned and captured using a light Olympus BX60 microscope with a digital camera (Spot camera, Diagnostic Instruments Inc.). Image J software was used to determine demyelination percentage and infiltration intensity. Also, in order to better recognize infiltrating cells on tissue section images, a protocol using image J software was designed to eliminate background tissue and to count infiltrated cells in Spinal Cord (SC)^22^. In summary, the protocol includes these stages: (*1*:*File* → *open image 2*:*Image* → *Adjust* → *Threshold*, *change the value in brightness part*: *upper value* = *113*, *Lower value* = *225*, *Thresholding method* = *Default*, *Threshold Color* = *White*, *Color space* = *HSB*, *check dark background*, *close the windows*. *3*:*Process* → *Noise* → *remove outliers*, *Change the value Radius* = *5*.*0*, *Threshold* = *50*, *which outliers* = *Dark and ok*. *4*:*process* → *smooth*, *repeat this stage to get a clearer picture*). The infiltrated cells were counted (7 sections/group, 4 fields/section) and results reported as mean number of cells per field per group.

#### Immunohistochemistry

Following de-paraffinziation and hydration, sections were permeabilized by 20% tween for 20 minutes for fluorescence immunostaining. Non-specific labelling was blocked with 0.1% BSA in 0.1% Triton X-100/PBS for 60 minutes. Sections were incubated overnight at 4 °C with primary antibodies anti-MBP (1:500) and anti-GFAP (1:800). Then, the slides were incubated with appropriate secondary antibodies (Alexa Fluor 488 and 566, 1:500; and Hoechst, 1:1000) for 1 hour. All antibodies were obtained from Abcam, USA. Samples were analyzed using a fluorescent microscope (Olympus IX-71; Olympus, Tokyo, Japan) equipped with a Canon EOS digital camera^22^.

#### Quantifying serum cytokines by enzyme-linked immunosorbent assay

After sacrifice, blood samples were collected from all rats via cardiac puncture. Serum was obtained following centrifugation (2500 rpm, 10 min), and frozen at −80 °C until ELISA test was performed. Serum levels of IL-4 and IFN-γ were measured using ELISA Kit (Sigma Aldrich, USA) according to manufacturer’s protocol.

#### Assessment of serum lactate by High Performance Liquid Chromatography (HPLC)

The chromatographic measurements of serum lactate were carried out with a KNAUER smartline High Performance Liquid Chromatography system (HPLC) equipped with micro vacuum degasser, LPG system, UV-VIS Detector (2550 was set at 220 nm) and a MZ ODS-C18 (250 mm × 4.6 mm, 5 μm) column. The chromatographic calculations were performed using an EZCHROM elite system. Determination of lactate was performed by HPLC according to the method described by Artiss *et al*.^82^ at the optimum separation condition with isocratic binary mobile phase consisting of 30:70 (v/v) of methanol: phosphate buffer (0.1 M) with a flow rate of 1 mL min^−1^. A digital pH meter (InoLab pH 730, Germany) was employed for pH measurements. A HERMLE bench centrifuge model 2206A (Germany) was used to accelerate the phase separation. The accuracy and applicability of the proposed method for the extraction and determination of lactate in serum were investigated using standard addition method.

#### Statistical analysis

Results are presented as an average with error bars indicating the standard error of the mean (Mean ± SEM). GraphPad Prism (Version 6.01, San Diego, CA, USA) software was used to perform statistical analyses. Ordinary one-way ANOVA followed by Tukey’s multiple comparison test, with a single pooled variance was used to analyze the data. Significance is indicated by *p < 0.05; **p < 0.01; ***p < 0.001 and ****p < 0.0001.
